# The impact of replacing dairy products with plant-based dairy alternatives on iodine and other micronutrients: a modelling study and evaluation of policy options to support population intake

**DOI:** 10.1016/j.lanplh.2026.101515

**Published:** 2026-11-04

**Authors:** Katie Nicol, Anne P Nugent, Jayne V Woodside, Kathryn H Hart, Sarah C Bath

**Affiliations:** a Discipline of Nutrition, Exercise, Chronobiology and Sleep, Faculty of Health and Medical Sciences, University of Surrey, Surrey, UK; b Institute for Global Food Security, School of Biological Sciences, Queen’s University Belfast, Belfast, UK; c Institute of Food and Health, School of Agriculture and Food Sciences, University College Dublin, Dublin, Ireland; d Centre for Public Health, School of Medicine, Dentistry and Biomedical Sciences. Queen’s University Belfast, Belfast, UK

## Abstract

**Background:**

Consumption of plant-based dairy alternatives (PBDAs) has increased as populations adopt more sustainable diets. However, PBDAs are often not fortified with key nutrients found in dairy products, particularly iodine. Iodine is essential for thyroid function and neurodevelopment, with increased importance during pregnancy, infancy, and adolescence. Iodine is often supplemented to cattle via feed, resulting in dairy products being a rich source. We assessed the impact of partly replacing dairy products with PBDAs on micronutrient intake and whether mandatory fortification of PBDAs or bread could mitigate potential shortfalls.

**Methods:**

This modelling study combined UK national dietary data with market data on PBDA fortification. Dairy-product intake was partly or fully replaced with commercially-available PBDAs on a gram-for-gram basis, aligned with Net Zero and EAT–*Lancet* targets (20–100% replacement). Usual nutrient intakes were estimated using the National Cancer Institute method, and the effects on nutrient intake, adequacy, and risk of excess were evaluated under current and mandatory fortification scenarios.

**Findings:**

Even partial replacement of dairy products with commercially-available PBDAs reduced iodine, calcium, riboflavin, and vitamin B12 intakes, particularly among young children, adolescents, and adult females. Replacement that aligned with the EAT–*Lancet* targets reduced iodine intake by 13–28%. Mandatory PBDA fortification prevented declines in micronutrient intake, although iodine inadequacy remained high among adolescent females (59% inadequate). Iodine-fortified bread improved iodine intake, reducing inadequacy among adolescent females to 37%. The combination of mandatory iodine fortification of both PBDAs and bread was the most effective strategy, maintaining or improving iodine adequacy across all population groups. There was no risk of excess nutrient intake.

**Interpretation:**

Partial replacement of dairy products with PBDAs can reduce micronutrient intake under current market fortification practices, especially among population groups already at risk of iodine deficiency. However, fortification of PBDAs, bread, or both represents a feasible evidence-based strategy to counteract these reductions in micronutrient intakes, minimise the impact on population health, and inform national nutrition policy and fortification strategies during transitions to more sustainable diets.

**Funding:**

Biotechnology and Biological Sciences Research Council FoodBioSystems.

## Introduction

Achieving sustainable and resilient food systems within planetary boundaries requires urgent, large-scale dietary changes while ensuring nutritious diets for a growing global population.^1^ The EAT–*Lancet* Commission^2^ recommends reducing animal-source food consumption, particularly in high-income countries, to mitigate environmental impact and promote sustainable food production. In countries such as the UK, where animal-source food consumption remains high,^3^ these recommendations would require substantial dietary adjustments. The UK Climate Change Committee has incorporated such targets into its Net Zero strategy, which includes a 20% reduction in meat and dairy consumption by 2030 and a 35% reduction by 2050.^4^ These targets reflect the growing recognition that dietary change is essential for achieving climate objectives.^2^^,^^4^ However, maintaining adequate intake of essential nutrients traditionally provided by animal-source products remains a key challenge during this transition. Although dairy products are the main source of several essential micronutrients, including calcium, vitamin B12, and iodine, they contribute substantially to greenhouse gas emissions.^2^ Reducing dairy product consumption aligns with sustainability targets but raises concerns about maintaining adequate micronutrient intake.

**Research in context**

**Evidence before this study**

We searched PubMed from inception to July 21, 2025, using the terms (“iodine” AND “dietary modelling” AND “plant-based” AND “dairy” OR “milk”), and identified four relevant studies. Existing research indicates that plant-based dairy alternatives (PBDAs) generally contain lower levels of essential micronutrients, particularly iodine, than dairy products. Iodine is essential for thyroid function and neurodevelopment, with increased importance during pregnancy, infancy, and adolescence. Most dietary modelling studies have focused primarily on plant-based drinks, offering minimal insight into the broader impact of PBDAs on iodine intake. In addition, these studies have largely focused on adult populations, overlooking vulnerable groups such as children and adolescent females, who are already at increased risk of iodine deficiency. The UK, which does not have a formal salt-iodisation policy, relies heavily on dairy products for iodine intake, with milk iodine concentrations influenced by iodine supplementation of cattle feed for animal health and the use of iodine-containing disinfectants in dairy production. The UK may therefore be particularly vulnerable to dietary shifts towards PBDAs. Consequently, a substantial evidence gap remains regarding how shifts towards PBDAs affect iodine intake and overall micronutrient adequacy.

**Added value of this study**

To our knowledge, this study presents the first UK-specific modelling of the impact of replacing milk, yoghurt, and cheese with PBDAs, using nationally-representative dietary data and market-specific fortification information. We simulated both partial and complete replacement scenarios aligned with the UK’s Net Zero and EAT–*Lancet* dietary targets and assessed nutrient adequacy and the risk of excess intake across age and sex subgroups. Our findings show that iodine intake is highly sensitive to even partial replacement of dairy products with commercially-available PBDAs, particularly among women and children. We further show that mandatory fortification of PBDAs or bread with iodine could prevent widespread iodine inadequacy without increasing the risk of excess iodine intake. This study provides a transferable modelling framework that could be adapted for use in countries with similar dietary patterns and food fortification practices that can inform national nutrition policy and fortification strategies during transitions to more sustainable diets.

**Implications of all the available evidence**

The global shift towards sustainable diets should consider the risk of micronutrient deficiencies arising from the replacement of nutrient-rich animal-sourced foods. In settings without salt iodisation, increased reliance on PBDAs could undermine iodine sufficiency in the absence of supportive policies. Although current fortification of PBDAs is insufficient to safeguard iodine intake, strategic interventions such as mandatory fortification of PBDAs, staple foods, or both, offer practical and effective solutions. This study, together with international evidence on successful iodine fortification programmes, underscores the importance of aligning nutrition policy with environmental targets. Nutrition policy should evolve alongside climate targets to ensure that shifts towards more sustainable diets do not compromise public health. Updating dietary guidelines, fortification standards, and labelling regulations will be crucial for maintaining micronutrient adequacy during the transition to more sustainable diets. Tailored policies addressing dietary guidelines, fortification standards, and labelling regulations will help to ensure that sustainable diets remain nutritionally adequate and environmentally sustainable, thereby aligning global sustainability targets with public health needs.

Plant-based dairy alternatives (PBDAs) offer a convenient means of reducing dairy product consumption without requiring major dietary changes, making PBDAs an increasingly popular choice among individuals trying to reduce their intake of animal-source foods.^5^ Although PBDAs can mimic the appearance and functionality of dairy products, their nutritional composition differs substantially.^6^ In particular, the iodine content of PBDAs is often inadequate,^6^ as only 20% of plant-based milk alternatives are fortified, and iodine fortification is virtually absent from plant-based cheese and yoghurt alternatives.^6^ Even when these products are fortified, their iodine concentration is often lower than that of cow’s milk products.^6^ Consequently, replacing dairy products with PBDAs without additional dietary modifications could increase the risk of iodine deficiency.^7^ Observational and intervention studies have consistently shown that individuals following plant-based diets have lower iodine intakes and are at greater risk of iodine deficiency than omnivores.^8^^,^^9^ Given the essential role of iodine in thyroid function and cognitive development,^10^ insufficient iodine intake can have considerable health consequences, particularly during pregnancy and early life.^10^

The UK is particularly vulnerable to iodine insufficiency because it does not have an iodine fortification policy, such as universal salt iodisation.^11^ Unlike countries where iodised salt is widely available, dairy products make an important contribution to iodine intake in the UK. The iodine content of dairy products is influenced by iodine supplementation of cattle feed for animal health and the use of iodine-containing disinfectants in dairy production,^9^ contributing to their role as rich dietary sources of iodine. Evidence of suboptimal intake is already apparent in specific population groups, with mild deficiency reported among pregnant women and adolescent girls.^3^^,^^12^ Without alternative dietary sources of iodine, further reductions in dairy product consumption could exacerbate iodine insufficiency and have important public health implications.

As food systems transition towards sustainability, strategies are needed to mitigate the potential nutritional risks of reduced dairy product consumption. One promising approach is the fortification of alternative food sources. Bread, a staple food widely consumed across demographic groups,^3^ represents an effective vehicle for iodine fortification. Several countries, including Australia and New Zealand, have successfully implemented iodine fortification using iodised salt in bread, thereby improving population-level iodine intake without requiring major dietary changes.^13^, ^14^, ^15^ The UK also has a precedent for such initiatives. Since the 1940s, white flour has been fortified with calcium, iron, thiamine, and niacin to address nutrient deficiencies,^16^ showing the feasibility of using the food supply to support public health. Most recently, legislation was updated to mandate folic acid fortification of flour to prevent neural tube defects, further reinforcing the UK’s commitment to targeted nutrient interventions through staple foods. Incorporating iodine (through iodised salt) into bread could provide a scalable and effective strategy for maintaining iodine intake, particularly as plant-based diets become more widely adopted.

We evaluated the impact of reducing dairy product intake, in line with UK Net Zero and EAT–*Lancet* targets, on population micronutrient (iodine, calcium, riboflavin, and vitamin B12) intakes using nationally-representative dietary data. These micronutrients were selected based on the contribution of dairy foods to overall micronutrient intake and their inclusion in PBDA fortification practices in the UK.^6^^,^^17^ We further assessed whether mandatory fortification strategies could mitigate potential reductions in micronutrient intake. Mandatory fortification of PBDAs was modelled for all four micronutrients at industry-standard fortification levels. In addition, bread was evaluated as an alternative plant-based vehicle for iodine fortification, reflecting the greater increase in iodine inadequacy observed under dairy replacement scenarios. Moreover, a combined scenario including mandatory fortification of both PBDAs and bread with iodine was modelled.

## Methods

### Study population and dietary assessment

The present study used food intake data from the National Diet and Nutrition Survey (NDNS) Rolling Programme (Years 9–11, 2016–19), obtained from the UK Data Archives.^3^ The NDNS includes individuals aged 1·5–100 years. We included data from children and adults across all available age groups (n=3558). To account for day-to-day variations in dietary intake, usual intake distributions were estimated using the National Cancer Institute method.

Participants whose food diaries contained no dairy products across all four recording days were excluded from the study, because dairy replacement scenarios could not be applied. Participants who reported at least one day of dairy intake were retained. Overall, 87% (n=3095) of participants reported consuming dairy products at least once and were included in the analysis (appendix p 4).

### Food categorisation to identify dairy products and dairy-containing foods

All food, beverage, and recipe codes in the NDNS food file containing milk, cheese, or yoghurt were identified and labelled (eg, skimmed, semi skimmed, whole milk, cow’s milk cheese, flavoured, and unflavoured yoghurts).^18^ Codes excluded from the scenarios were dried milk, plant-based alternatives, and follow-on milk. The contribution of milk, cheese, and yoghurt to relevant recipes was calculated using the Food Standards Agency standard recipes database.^18^

As the NDNS food records do not consistently capture brand-level information, foods were categorised into dairy groups (milk, cheese, and yoghurt) using food codes. For composite foods, the contribution of dairy ingredients (milk, cheese, and yoghurt) was quantified using the Food Standards Agency standard recipes database.^18^ Only the dairy component of these foods was replaced in the modelling. Plant-based alternatives were then assigned probabilistically at the product level within each category, with each product assigned an equal probability of selection to capture variation in micronutrient composition across products.

### Market survey micronutrient concentration data

The methods used to identify retail PBDAs have been previously reported.^6^ Briefly, plant-based alternatives to milk, yoghurt, and cheese were identified through product searches of UK supermarket websites conducted in December, 2020. Micronutrient concentrations were obtained from product labels, information provided on supermarket and manufacturers’ websites, or product nutritional databases.

### Dairy product replacement scenarios

We modelled the impact of dietary replacement scenarios on iodine, calcium, riboflavin, and vitamin B12 intakes by replacing milk, cheese, and yoghurt consumed by the UK population with PBDAs available in UK supermarkets.^6^ The scenarios were designed to reflect 2019 recommendations regarding animal-source food consumption. To achieve the UK’s Net Zero targets, the UK Climate Change Committee recommends reducing total animal product intake by 20% by 2030 and 35% by 2050.^4^ Additionally, the EAT–*Lancet* Commission recommends a dietary shift that includes a greater than 50% reduction in the global consumption of animal-based foods, such as red meat and dairy foods.^2^ Therefore, we used four levels of dairy replacement: 20%, 35%, 50%, and 100%. Under these scenarios, the corresponding amount of dairy milk, cheese, and yoghurt in a participant’s diet was replaced with PBDAs (table). Replacement was conducted on a gram-for-gram basis, whereby the weight of dairy foods consumed was directly substituted with an equivalent weight of plant-based alternatives. To mitigate losses in iodine intake due to the shift towards PBDAs, we considered three policy options: mandatory fortification of PBDAs, mandatory fortification of bread, or both (appendix p 24).

**Table:** Scenarios used to model micronutrient intakes at varying levels of dairy-product replacement with plant-based dairy alternatives (PBDA)

	Micronutrient concentrations in PBDAs				Proportion of PBDAs fortified (%)				Iodised salt in bread (mg/kg)
	Iodine (μg/100 g)	Calcium (mg/100 g)	Riboflavin (mg/100 g)	Vitamin B12 (μg/100 g)	Iodine	Calcium	Riboflavin	Vitamin B12	
1. Current market fortification	0–45	0–189	0–0·75	0–0·94	12	61	19	46	0
2A. Mandatory PBDA fortification	22·5	120	0·21	0·38	100	100	100	100	0
2B. Mandatory bread fortification	0–45	NA	NA	NA	12	NA	NA	NA	30
3. Comprehensive mandatory fortification	22·5	NA	NA	NA	100	NA	NA	NA	30

Scenario 1: Partial replacement of dairy products with PBDAs based on existing UK market fortification strategies (current market fortification). Scenario 2A: Partial replacement of dairy products with PBDAs, assuming mandatory fortification of PBDAs with iodine, calcium, riboflavin, and vitamin B12 of PBDAs at an industry-standard level (mandatory PBDA fortification). Scenario 2B: Partial replacement of dairy products with PBDAs based on existing fortification strategies, with mandatory iodine fortification of bread (mandatory bread fortification). Scenario 3: Partial replacement of dairy products with PBDAs, assuming mandatory iodine fortification of both PBDAs and bread (comprehensive mandatory fortification). Ranges reflect the distribution of micronutrient concentrations across products identified in the UK market survey.^6^ PBDA=plant-based dairy product alternative.

Mandatory fortification of PBDAs was modelled for iodine, calcium, riboflavin, and vitamin B12, reflecting nutrients commonly added to these products in the UK. For bread, only iodine fortification was modelled. Bread was selected as an alternative plant-based vehicle because it is widely consumed and has been used as a fortification vehicle in other countries.^13^, ^14^, ^15^ This approach also reflects existing UK policy, under which wheat flour is already mandatorily fortified with calcium.

Each instance of dairy product consumption in the NDNS dietary records was replaced using a probabilistic assignment approach in which a PBDA was selected at the individual product level. Within each category (milk, cheese, and yoghurt), products identified in the market survey were assigned an equal probability of selection, thereby allowing variability in micronutrient composition across products to be reflected in the modelling.

#### Scenario 1: Partial replacement of dairy products with PBDAs based on existing UK market fortification strategies (current market fortification)

This scenario reflects the existing UK market,^6^ wherein the fortification of PBDAs is voluntary. To capture variations in fortification practices, we incorporated market survey data^6^ to account for the probability of PBDAs being fortified and the range of micronutrient fortification concentrations reported on product labels. These fortification ranges reflect the distribution of micronutrient concentrations observed across products in the UK market survey.^6^ In the modelling, product-level micronutrient concentrations were incorporated probabilistically, allowing the full variation in fortification levels across products to be represented.

#### Scenario 2A: Partial replacement of dairy products with PBDAs, assuming mandatory fortification of PBDAs (mandatory PBDA fortification)

In this scenario, we modelled the impact of a mandatory policy requiring all PBDAs to be fortified with iodine, calcium, riboflavin, and vitamin B12 at industry-standard fortification levels. Fortification levels were based on the most observed concentrations in the UK market^6^ and correspond to the minimum levels required for products to be labelled as a source of each micronutrient (table).

#### Scenario 2B: Partial replacement of dairy products with PBDAs based on existing fortification strategies, with mandatory iodine fortification of bread (mandatory bread fortification)

In this scenario, we modelled the replacement of dairy products with PBDAs under current market fortification practices (Scenario 1), alongside mandatory iodine fortification of bread. All salt used in commercially produced bread products was replaced with iodised salt (30 mg iodine/kg salt) in line with WHO recommendations.^19^ Existing UK fortification practices (eg, calcium fortification of wheat flour) were retained in the analysis, because these practices are already included in the underlying food composition data. Sensitivity analyses were conducted using iodine concentrations across WHO-recommended range for salt iodisation to assess the impact of this assumption (appendix pp 18–23).

#### Scenario 3: Partial replacement of dairy products with PBDAs, assuming mandatory iodine fortification of both PBDAs and bread (comprehensive mandatory fortification)

This scenario combined both policy interventions by modelling the replacement of dairy products with PBDAs under Scenario 2A (ie, mandatory fortification of PBDAs with iodine) along with mandatory iodine fortification of bread at WHO-recommended concentrations (30 mg iodine/kg salt; Scenario 2B).^19^

### Data analysis

To characterise current milk, cheese, and yoghurt intakes in the NDNS, intake estimates were generated from individual dietary records detailing the food items consumed by each survey participant on each survey day. Estimated intake values (Mean, SE) represent projected 4-day averages for each participant. Usual nutrient intake distributions were estimated using the NCI method, which accounts for within-person variation in repeated dietary measures. To control for any selection bias in the associations with nutrient intake, NDNS survey weight factors were applied for individuals in each NDNS year.^3^

All modelling scenarios were conducted using the web-based software DaDiet© (Dazult, Version 17.04). The NCI method was used to estimate usual nutrient intakes and assess the percentage of the population meeting the threshold for adequacy.^20^ We used the UK estimated average requirement (EAR) to estimate the prevalence of inadequate intake and the UK upper limit to estimate the prevalence of excess intake. In nutritionally adequate populations, the proportion of individuals with intakes below the EAR is expected to be low (approximately 2–3%). As the UK does not have an EAR for iodine, we used Institute of Medicine values. We also report intakes below the UK lower reference nutrient intake, which is the intake level estimated to meet the requirements of only 2·5% of the population (or in the case of iodine, the intake required to prevent goitre) and commonly used as an indicator of a high risk of inadequacy. When dietary reference values or upper limits did not match the age bands used in this study, we averaged the values for two adjacent age groups. The UK lower reference nutrient intake, EAR, and upper limit thresholds used in this analysis are presented in the appendix (p 3).

Given that differences in modelling scenarios were introduced by design, typical statistical testing was not appropriate. Thus, to assess meaningful differences in the mean daily intakes between modelling scenarios, we examined the means and their 95% CIs. Non-overlapping CIs were considered to indicate meaningful differences. This approach has been used previously in dietary pattern studies.^21^^,^^22^

### Role of the funding source

The funder of the study had no role in study design, data collection, data analysis, data interpretation, manuscript writing, or decision to submit for publication.

## Results

Overall, 3095 (87%) of the total population of 3558 participants in the NDNS consumed at least one dairy product during the survey, with a mean intake of 237 g/day. The highest dairy product consumption was observed among male participants aged 1–3 years (150 (92%) of 164 participants; 395 g/day), whereas female participants aged 14–18 years had the lowest intake (164 (76%) of 217 participants; 162 g/day). Milk was the largest contributor to total dairy product intake, accounting for 85% of total consumption. However, adolescent female participants and women of reproductive age (19–49 years) consumed less milk than the total population (168 g/day for adolescent female participants, 163 g/day for women of reproductive age, and 215 g/day for the total population). Bread was consumed by 2134 (60%) of 3558 participants, with a mean intake of 93 g/day (approximately two slices). The total dairy product intake, dairy product subgroups (milk, cheese, and yoghurt), and bread intake for the total population and separate population subgroups before dietary modelling are presented in the appendix (p 4).

Iodine intake was generally adequate across much of the total population (appendix pp 5–12). However, some vulnerable groups, such as adolescent female participants, had inadequate iodine intakes from their usual diet. Among female participants aged 14–18 years, 117 (54%) of 217 participants had inadequate iodine intake, with a mean intake of 97 μg/day (appendix pp 9). Calcium inadequacy was also high in this group, affecting 117 (54%) of 217 individuals, with a mean intake of 641 mg/day (appendix p 15).

The partial replacement of dairy products with PBDAs under current UK market fortification strategies (Scenario 1) resulted in notable declines in micronutrient intakes. The largest reductions were observed among young children, adolescents, and adult females. Replacing 35% of dairy product consumption with PBDAs resulted in a 9–21% decrease in the mean daily iodine intake relative to the baseline intake, and replacing 50% of dairy product consumption with PBDAs resulted in a 13–28% decrease in the mean daily iodine intake across most population groups. Among adolescents, iodine inadequacy increased when dairy product replacement reached at least 50%, rising to 72% in males and 80% in females at 100% replacement (figure 1; appendix pp 9). For females aged 19–49 years, the mean iodine intake declined at 35%, 50%, and 100% replacement, leading to increased iodine inadequacy (figure 1; appendix pp 10). Although the mean intakes of calcium, riboflavin, and vitamin B12 also declined at 35% and 50% replacement (figure 2), the proportion of the population with inadequate intakes only increased at higher levels of dairy product replacement (ie, 50% or 100%).

**Figure 1:**
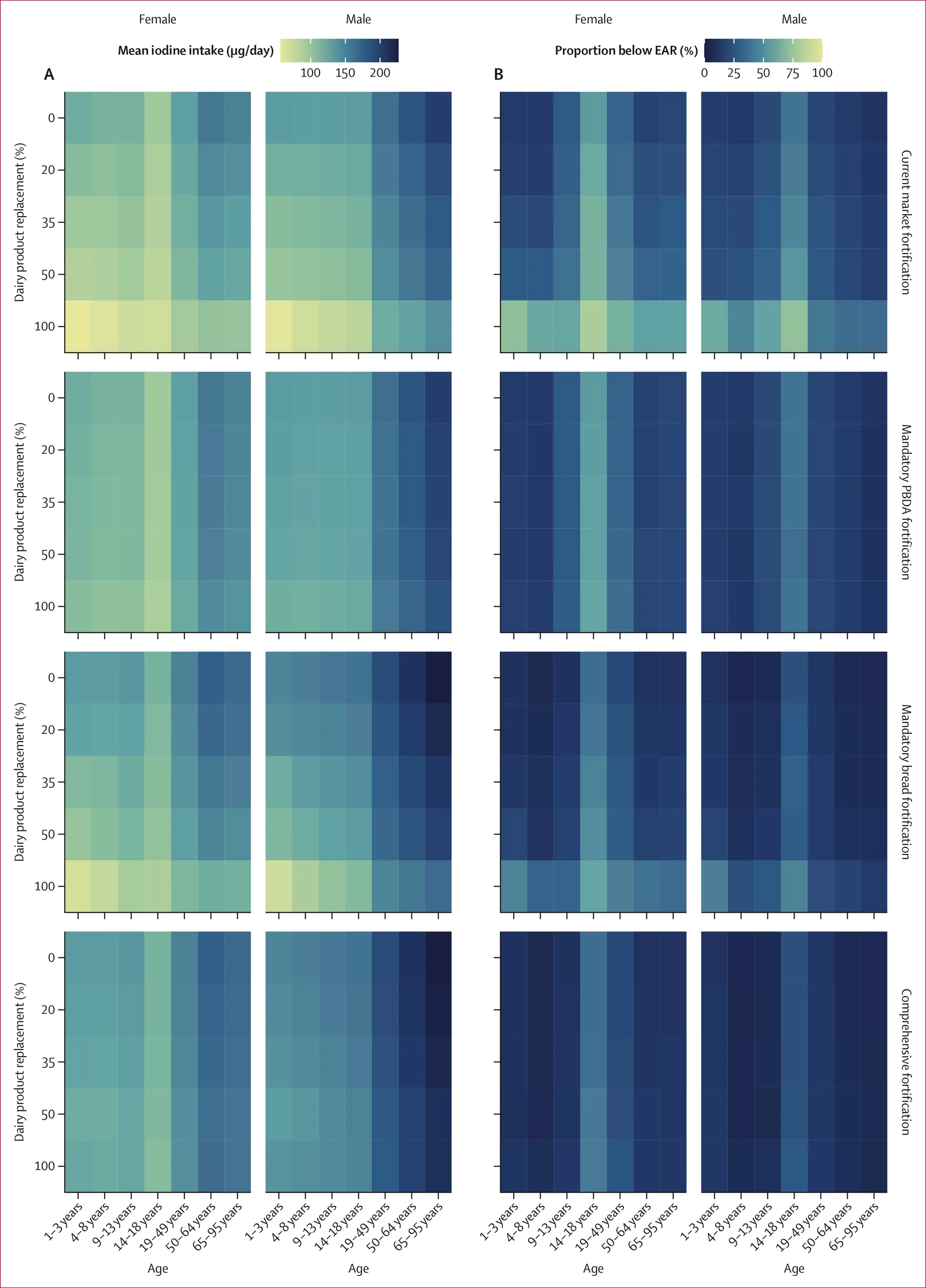
Effect of dairy replacement with plant-based alternatives and iodised bread on iodine intake and inadequacy Mean iodine intake (μg/day; A) and proportion of the population with inadequate iodine intake (defined as intake below the estimated average requirement) under partial or complete replacement of dairy milk, cheese, and yoghurt with PBDAs or the addition of iodised salt to bread (B). The results are stratified by sex and age group. EAR=estimated average requirement. PBDA=plant-based dairy alternative.

**Figure 2:**
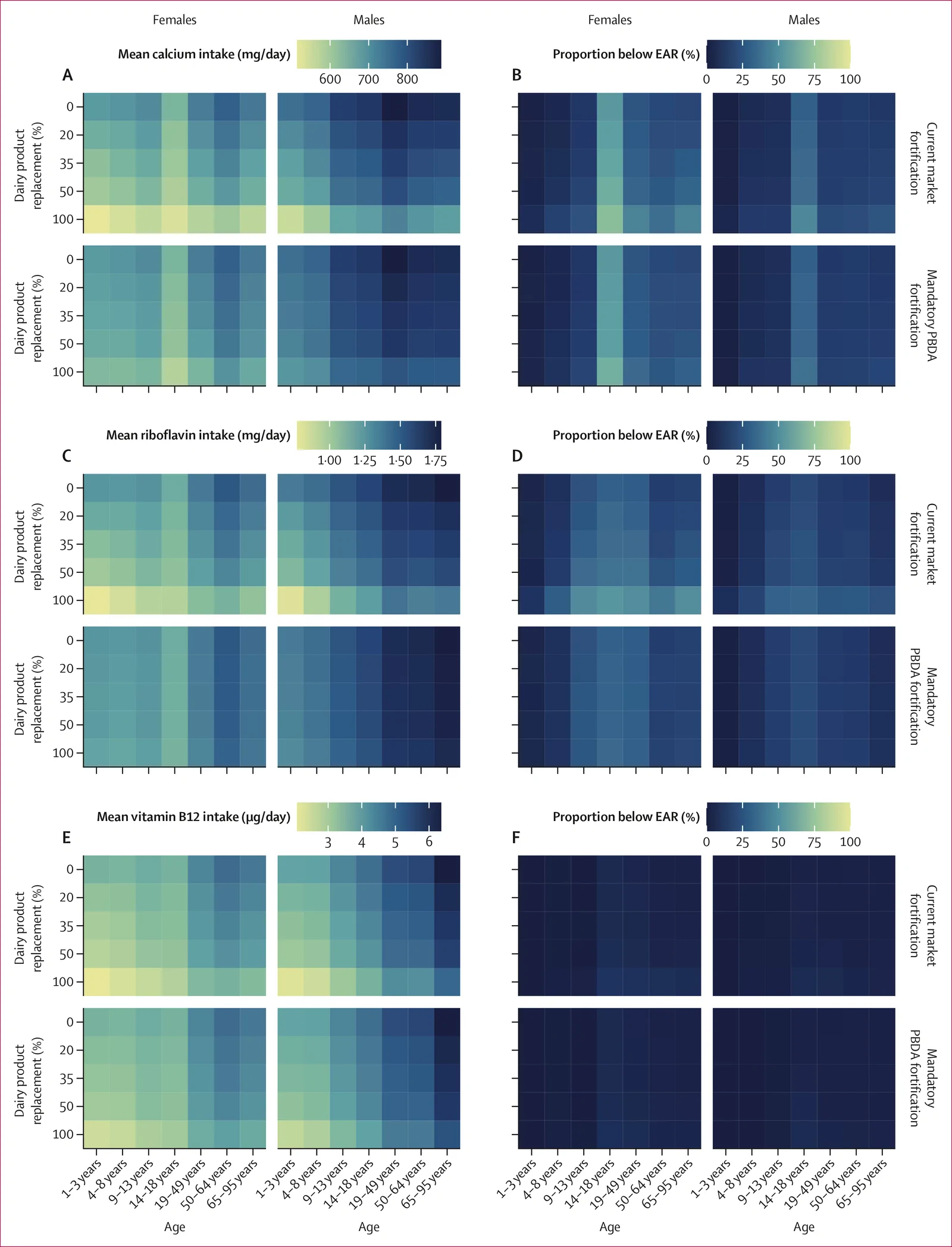
Effect of dairy replacement with plant-based alternatives on calcium, riboflavin, and vitamin B12 intake and inadequacy (A) Mean calcium intake. (B) Proportion of population with inadequate calcium intake (defined as intake below the estimated average requirement). (C) Mean riboflavin intake. (D) Proportion of population with inadequate riboflavin intake. (E) Mean vitamin B12 intake. (F) Proportion of population with inadequate vitamin B12 intake. Results are stratified by age group and sex. EAR=estimated average requirement. PBDA=plant-based dairy alternative.

When PBDAs were mandatorily fortified with iodine, calcium, riboflavin, and vitamin B12 (Scenario 2A), no changes in mean intakes were observed at any level of dairy product replacement in any population group compared with the usual diet (figures 1 and 2). Iodine, calcium, and riboflavin intakes remained unchanged, whereas minor reductions in vitamin B12 intake were detected in some groups. Nonetheless, these reductions did not increase the prevalence of inadequate intake (figure 2). The prevalence of iodine inadequacy remained high among at-risk groups, particularly females aged 14–18 years (56–59% had intakes below the EAR) and 19–49 years (32–37% had intakes below the EAR; appendix pp 5–12).

The introduction of iodine-fortified bread (with iodised salt) improved population iodine intake (Scenario 2B), particularly at low levels of dairy product replacement (figure 1). Nevertheless, the proportion of the population with inadequate iodine intake at higher levels of dairy product replacement (ie, 50% or 100%). Among females aged 14–18 years, iodine inadequacy declined from 54% at baseline to 37% following the addition of iodised salt to bread (0% dairy replacement) (figure 1). Even at 20% and 35% dairy replacement, the mean iodine intake remained higher than that at baseline in this age group. Among females aged 19–49 years, iodine inadequacy decreased from 32% at baseline to 24% at 20% replacement (appendix pp 5–12). However, as dairy product replacement increased concurrently, iodine inadequacy also increased, reaching 44% at 100% replacement.

Sensitivity analyses showed minimal differences in estimated iodine intake and the prevalence of inadequacy across WHO-recommended range of salt iodisation levels, indicating that the findings were robust to plausible variation in fortification levels (appendix pp 18–23).

The most effective strategy for maintaining dietary iodine intake was a comprehensive fortification approach that combined mandatory iodine fortification of both PBDA and bread (Scenario 3). This approach successfully maintained or improved iodine adequacy across all age groups, even when dairy products were fully replaced (figure 1). In contract to Scenario 2B, where iodine inadequacy increased progressively as dairy replacement increased, Scenario 3 largely prevented this deterioration. Among females aged 14–18 years, iodine inadequacy declined from 54% at baseline to 37% at 20% dairy replacement and remained lower than baseline at higher replacement levels, reaching 43% at 100% replacement. The mean iodine intake also remained above baseline values across all replacement levels, increasing to 108 μg/day at 100% replacement. Among females aged 19–49 years, iodine inadequacy declined to 20% (from 32% at baseline) at 20% dairy replacement; the proportion with iodine inadequacy remained stable (at 21–22%) at 35% and 50% dairy replacement. Notably, the proportion of the population that exceeded the upper limit remained low and did not increase compared with the baseline levels (appendix pp 5–12).

## Discussion

Achieving Net Zero targets is essential for reducing the environmental impact of the food system. However, the nutritional consequences of replacing dairy products with PBDAs remain poorly understood. Our findings suggest that even a partial transition towards PBDAs, in line with the Net Zero^4^ and EAT-*Lancet*^2^ targets, could lead to declines in micronutrient adequacy, particularly of iodine. In the absence of effective policy interventions, these declines could lead to widespread micronutrient inadequacies among populations already at risk.

Even modest replacements of dairy products with PBDAs led to marked declines in iodine intake in vulnerable groups. Under current market fortification practices (Scenario 1), the prevalence of iodine inadequacy increased from 54% at baseline to 65% at 35% dairy replacement among adolescent female participants. Similarly, among women aged 19–49 years, iodine inadequacy increased from 32% at baseline to 42% at 35% dairy replacement. At 100% replacement, the prevalence of iodine inadequacy increased to 80% among adolescent females and 72% among adolescent males. Although the mean intakes of calcium, vitamin B12, and riboflavin also declined with increasing PBDA replacement, iodine was the most sensitive micronutrient, with iodine inadequacy increasing even at moderate levels of replacement. These findings indicate that, in the absence of appropriate policy measures, population-wide dietary shifts towards PBDAs might undermine progress towards micronutrient sufficiency. Addressing these nutritional risks through appropriate fortification policies is essential to support equitable and sustainable diets and to safeguard progress towards sustainable development goals (SDGs), particularly those related to improved nutrition (SDG 2) and health (SDG 3).^23^

Globally, universal salt iodisation is a WHO-recommended strategy for preventing iodine deficiency. Universal salt iodisation is the most widely implemented and effective public health intervention for controlling iodine deficiency, with current programmes estimated to have eliminated around 3·3 billion cases of inadequate iodine intake worldwide.^24^ However, iodised salt has never been widely implemented in the UK,^25^ where iodine sufficiency has historically been achieved through dairy consumption rather than through a formal fortification policy.^26^ Consequently, iodised salt remains minimally available^25^ and is rarely used in food manufacturing, contributing negligibly to population iodine intake.^25^ Implementing universal salt iodisation in the UK would therefore require substantial changes to food production practices and public health messaging, particularly in the context of longstanding salt-reduction strategies.^27^

Our results suggest that different policies might have greater benefits for specific population subgroups. Mandatory iodine fortification of PBDAs would be particularly beneficial for young children, who rely heavily on dairy products as a primary iodine source. The addition of iodised salt to bread could substantially improve iodine intake among young women, who already have the lowest iodine intake of any group (97 μg/day). Adding iodised salt to bread improved iodine intake by 9–40% in key subgroups, reducing inadequacy in adolescent females from 54% to 37% at 20% dairy product replacement. When both PBDA and bread fortification were implemented, this combined strategy offered a comprehensive and complementary policy response that maintained iodine adequacy across all population subgroups, even at 100% dairy product replacement.

The dietary modelling approach used in this study accounts for usual intake distributions, current consumption patterns, voluntary and mandatory fortification scenarios, and population heterogeneity. By applying this framework to the UK context, we show that current market fortification practices are insufficient to safeguard micronutrient intake during dietary transitions to include PBDAs rather than dairy. Although some PBDAs in the UK are fortified with calcium or vitamin B12, iodine fortification remains particularly rare, with only 12% of PBDAs containing added iodine.^6^ Our findings are consistent with those of a dietary modelling conducted in France,^28^ where replacing cow’s milk with PBDAs increased the risk of iodine deficiency among adults. Although no iodine-fortified PBDAs were available in the French setting, our results show that the iodine fortification used in UK products is inadequate to protect iodine status if PBDA consumption increases substantially.

In this context, and in the absence of an established universal salt iodisation programme, fortification of widely consumed staple foods such as bread represents a practical and scalable strategy for supporting micronutrient adequacy alongside environmental targets. Countries such as Australia, New Zealand, and the Netherlands^13^, ^14^, ^15^ have already shown the effectiveness of using iodised salt in bread, improving iodine status by 38–66% among children and adults.^13^^,^^14^ However, risk assessments conducted in countries such as Norway have highlighted potential concerns, with 8–18% of young children exceeding the upper limit for iodine.^29^ In our study, a similar proportion of children aged 1·5–3 years (14–23%) exceeded the upper limit when iodised salt was added to bread. Notably, even at baseline, 9–20% of children in this age group already exceeded the upper limit, highlighting that this finding is not solely attributable to the addition of iodised salt. Nonetheless, the intervention did not increase the proportion of any population subgroup exceeding the upper limit. Moreover, some populations have tolerated higher iodine intakes over long periods without adverse effects, suggesting that the current upper limit, particularly those extrapolated from adult data, might be overly conservative when applied to children.^14^^,^^30^

The implications of our findings extend beyond the UK. Although product availability, regulations, and dietary patterns vary internationally, the modelling framework presented here offers a transferable approach for evaluating the nutritional implications of sustainable dietary transitions in other countries. By incorporating national food consumption and composition data, this framework can inform evidence-based nutrition policy worldwide. The thresholds used for mandatory fortification of PBDAs in our analysis align with the EU criteria for labelling a product as a source of micronutrients, ensuring relevance and feasibility within existing regulatory frameworks. Similarly, the iodine concentrations modelled for bread fortification are based on levels already implemented in countries such as the Netherlands, Australia, and New Zealand and are consistent with WHO recommendations,^19^ further supporting the global applicability of this strategy.

Our analysis advances the literature on the implications of integrating PBDAs into diets in several ways. Previous research has focused primarily on milk alternatives.^28^ Nonetheless, we included a broader range of PBDA products (eg, yoghurts and cheeses) that are now commonly available in retail outlets, thereby reflecting more realistic dietary substitution patterns. Moreover, modelling usual nutrient intakes removes the intraindividual variation apparent in short-term dietary recalls, providing a more realistic distribution of long-term average intakes and improving the evaluation of population proportions against dietary reference values. An earlier study^28^ evaluating the partial replacement of milk with PBDAs used national dietary surveys as the reference diet, similar to our study; however, this study did not consider the distribution of intakes naturally present within a population, a key consideration for informing nutrition policy. Lastly, by explicitly modelling fortification strategies, albeit within the UK context, we provide a data-driven, policy-relevant framework that bridges the gap between sustainability targets and public health nutrition.

Our study has limitations. First, the study assumes a population-wide shift in dietary behaviour and broad acceptance of PBDAs, which might not fully reflect individual behaviours or preferences. Consumer acceptance of PBDAs varies widely according to age, sex, and sociocultural context.^31^^,^^32^ Although the PBDAs modelled are representative of current offerings, the market is evolving rapidly and product formulations might change. Second, in the absence of market share data, all PBDA products were assigned equal selection probabilities in the modelling, which might not reflect real-world consumer purchasing patterns. Third, our analysis did not address nutrient bioavailability or nutrient–nutrient interactions, which require further investigation to assess the full nutritional impact of integrating fortified PBDAs into diets. Fourth, the analysis was restricted to individuals reporting some dairy consumption (87% of the study population) and therefore does not capture the potential impact of fortification strategies among non-dairy consumers. These uncertainties underscore the importance of ongoing research and real-world monitoring of dietary patterns and nutrient intakes as the food system continues to evolve.

Despite these limitations, our findings provide quantitative evidence to inform ongoing policy discussions on reformulation standards and fortification strategies for PBDAs and staple foods in the UK. By providing quantitative evidence to inform policy discussions, this study extends beyond academic modelling by identifying actionable policy levers to improve population-level iodine intake, particularly in the context of dietary transitions towards more sustainable diets.

In conclusion, this study highlights an important nutrition policy challenge: aligning environmental sustainability with micronutrient adequacy. If left unaddressed, a shift towards plant-based diets could unintentionally exacerbate nutritional risks, particularly iodine deficiency in adolescent girls and women. Our findings indicate that current voluntary fortification practices are insufficient to support a healthy and equitable transition towards Net Zero and EAT–*Lancet* dietary targets. Mandatory fortification of PBDAs and the use of iodised salt in bread are effective, evidence-based strategies that can help to protect public health during this transition. Proactive policy action is essential not only in the UK but also globally to ensure that climate-aligned food systems do not compromise public health.

## Data sharing

The data underlying the results presented in the study are available from the UK Data Service: (https://beta.ukdataservice.ac.uk/datacatalogue/studies/study?id=8159), (https://beta.ukdataservice.ac.uk/datacatalogue/studies/study?id=6533).

## Declaration of interests

SCB has received an honorarium each from Oatly UK, Dairy UK, and Dairy Council NI for delivering lectures and webinars for health-care professionals. AN has received an honorarium from Dairy Council NI for delivering a lecture for health-care professionals. All other authors declare no competing interests.
