# Supplementary material for: The impact of replacing dairy products with plant-based dairy alternatives on iodine and other micronutrients: a modelling study and evaluation of policy options to support population intake
Source: Lancet Planet Health. 2026 Nov 4;10(9):None. doi: 10.1016/j.lanplh.2026.101515 (PMC13734843; doi:10.1016/j.lanplh.2026.101515)
Supplement: Supplementary file 1 [file mmc1.pdf]

THE LANCET
Planetary Health

Supplementary appendix

This appendix formed part of the original submission and has been peer reviewed. We post it as supplied by the authors.

Supplement to: Nicol K, Nugent AP, Woodside JV, et al. The impact of replacing dairy products with plant-based dairy alternatives on iodine and other micronutrients: a modelling study and evaluation of policy options to support population intake. *Lancet Planet Health* 2026. https://doi.org/10.1016/j.lanplh.2026.101515

Title: A modelling study of the impact of replacing dairy products with plant-based dairy alternatives on iodine and other micronutrients: an evaluation of policy options to support population intake

Authors: Katie Nicol[1], Anne P. Nugent[2,3], Jayne V. Woodside[2,4], Kathryn H. Hart[1], Sarah C. Bath[1]

Table of Contents

Supplementary Table 1. Participant characteristics of the NDNS years 9-11 (2016-2019) dataset stratified by age and gender. 2

Supplementary Table 2. Lower reference nutrient intake, estimated average requirement and upper limit thresholds used in the present analysis 3

Supplementary Table 3. Mean daily intakes of milk, cheese, yoghurt and bread (consumers only) in the NDNS years 9-11 (2016-2019) stratified by age and gender. .. 4

Supplementary Table 4. The impact of total or partial replacement of dairy milk, cheese, and yoghurt with plant-based alternatives and/or the addition of iodised salt to bread products on iodine intakes (µg/day) and % with intakes below the EAR and LRNI and above the UL 5

Supplementary Table 5. The impact of total or partial replacement of dairy milk, cheese, and yoghurt with plant-based alternatives on calcium (mg/day), riboflavin (mg/day) and vitamin B12 intakes and % with intakes below the EAR and LRNI 13

Supplementary Table 6. The impact of total or partial replacement of dairy milk, cheese, and yoghurt with plant-based alternatives and/or the addition of iodised salt to bread products (20 mg of iodine per kg of salt) on iodine intakes (µg/day) and % with intakes below the EAR and LRNI and above the UL. 18

Supplementary Figure 1. Overview of dietary modelling scenarios.......... 24

Supplementary Table 1. Participant characteristics of the NDNS years 9-11 (2016-2019) dataset stratified by age and gender.

Age	Gender	n
1.5-3 years	Male	164
	Female	142
4-8 years	Male	257
	Female	251
9-13 years	Male	260
	Female	231
14-18 years	Male	192
	Female	217
19-49 years	Male	358
	Female	554
50-64 years	Male	212
	Female	268
65+ years	Male	193
	Female	259
**Total population**		**3558**

Supplementary Table 2. Lower reference nutrient intake, estimated average requirement and upper limit thresholds used in the present analysis

		Iodine			Calcium		Riboflavin		Vitamin B12	
		LRNI	EAR	UL	LRNI	EAR	LRNI	EAR	LRNI	EAR
**1.5-3 years**	Male	40	65	200	200	275	0.2	0.5	0.3	0.4
	Female	40	65	200	200	275	0.2	0.5	0.3	0.4
**4-8 years**	Male	50	65	300	300	390	0.45	0.7	0.55	0.75
	Female	50	65	300	300	390	0.45	0.7	0.55	0.75
**9-13 years**	Male	60	73	450	400	450	0.65	0.9	0.7	0.85
	Female	60	73	450	400	450	0.65	0.9	0.7	0.85
**14-18 years**	Male	70	95	450	480	750	0.8	0.9	1.0	1.25
	Female	70	95	450	450	625	0.8	0.9	1.0	1.25
**19-49 years**	Male	70	95	600	400	525	0.8	1.0	1.0	1.25
	Female	70	95	600	400	525	0.8	1.0	1.0	1.25
**50-64 years**	Male	70	95	600	400	525	0.8	1.0	1.0	1.25
	Female	70	95	600	400	525	0.8	1.0	1.0	1.25
**65+ years**	Male	70	95	600	400	525	0.8	1.0	1.0	1.25
	Female	70	95	600	400	525	0.8	1.0	1.0	1.25

UK Estimated Average Requirements (EAR) was used to estimate the prevalence of inadequate intake for all nutrients except for iodine as the UK does not have an EAR for iodine so the IOM EAR was used. The Lower Reference Nutrient Intake (LRNI) was also used for iodine as that is the lower value used in the DRV report. The UK Upper Limit (UL) was to estimate the prevalence of excess intake. Where DRVs/UL do not match up with the age bands used in this study, we averaged two age groups.

Supplementary Table 3. Mean daily intakes of milk, cheese, yoghurt and bread (consumers only) in the NDNS years 9-11 (2016-2019) stratified by age and gender.

		**Total dairy products (g/day)**		**Milk (g/day)**		**Cheese (g/day)**		**Yoghurt (g/day)**		**Bread (g/day)**	
		% Consumers	Mean	% Consumers	Mean	% Consumers	Mean	% Consumers	Mean	% Consumers	Mean ± SD
**1.5-3 years**	Male	92	395	88	361	36	27	39	95	56	53
	Female	93	261	80	236	47	25	45	92	48	58
**4-8 years**	Male	90	269	75	258	32	37	36	101	66	81
	Female	88	270	77	254	32	27	32	100	65	75
**9-13 years**	Male	84	245	69	238	33	37	24	116	63	99
	Female	86	206	72	201	37	34	22	94	68	84
**14-18 years**	Male	75	297	60	313	31	50	10	168	68	123
	Female	76	162	54	168	31	36	15	121	64	92
**19-49 years**	Male	86	215	71	204	34	49	18	120	59	126
	Female	85	196	73	163	36	45	21	137	55	94
**50-64 years**	Male	85	239	82	210	28	43	14	134	56	122
	Female	91	225	82	171	40	44	33	132	52	81
**65+ years**	Male	95	260	90	227	38	41	20	145	65	97
	Female	96	218	88	175	30	42	34	112	55	70
**Total population**		87	237	75	215	34	40	25	116	60	93

Supplementary Table 4. The impact of total or partial replacement of dairy milk, cheese, and yoghurt with plant-based alternatives and/or the addition of iodised salt to bread products on iodine intakes (µg/day) and % with intakes below the EAR and LRNI and above the UL

		% replacement of dairy	Mean	5th	50th	95th	99th	% < LRNI	% < EAR	% > UL
**Males aged 1.5-3 years (n = 164)**	Usual diet	0%	134	45	116	260	295	3	16	20
	Scenario 1: current market	20%	118	42	107	228	259	4	19	10
		35%	**107***	38	**97***	**198***	**232***	7	21	**5***
		50%	**100***	34	**93***	**182***	**237***	9	23	**4***
		100%	**61***	**25***	**58***	**111***	**137***	**22***	**61***	**0***
	Scenario 2A: mandatory PBA fortification	20%	132	45	117	252	285	3	16	18
		35%	128	44	118	240	287	3	16	12
		50%	127	43	120	241	298	4	16	12
		100%	120	44	117	217	277	3	17	**7***
	Scenario 2B: mandatory bread iodisation	0%	146	58	133	267	300	1	10	23
		20%	141	55	128	263	296	2	13	23
		35%	121	53	113	219	250	2	13	10
		50%	**112***	47	105	**193***	229	3	19	**4***
		100%	**74***	35	**69***	**124***	**150***	11	**44***	**0***
	Scenario 3: comprehensive mandatory fortification	20%	144	56	135	267	295	1	12	21
		35%	140	56	132	253	295	1	12	16
		50%	137	52	129	255	288	1	12	16
		100%	132	53	129	227	287	1	12	10
**Females aged 1.5-3 years (n = 142)**	Usual diet	0%	121	48	107	238	295	4	15	9
	Scenario 1: current market	20%	108	45	98	206	260	4	17	6
		35%	**96***	43	**88***	179	227	4	22	2
		50%	**87***	39	**80***	**175***	**211***	6	27	2
		100%	**56***	**27***	**53***	**98***	**139***	**23***	**72***	**0***
	Scenario 2A: mandatory PBA fortification	20%	118	49	108	220	282	4	16	9
		35%	116	49	108	216	273	4	17	8
		50%	115	50	106	211	265	4	16	8
		100%	108	49	100	190	237	4	18	4
	Scenario 2B: mandatory bread iodisation	0%	133	57	124	246	313	3	10	14
		20%	129	53	119	243	313	3	10	11

		% replacement of dairy	Mean	5th	50th	95th	99th	% < LRNI	% < EAR	% > UL
		35%	111	49	104	193	248	3	13	4
		50%	**100***	47	**91***	181	229	3	19	3
		100%	**69***	36	**67***	**114***	**140***	11	**46***	**0***
	Scenario 3: comprehensive mandatory fortification	20%	131	57	124	230	306	3	10	13
		35%	128	60	122	222	270	3	10	11
		50%	125	54	118	221	289	3	10	10
		100%	121	56	115	201	239	2	9	6
**Males aged 4-8 years (n = 257)**	Usual diet	0%	132	48	117	265	342	7	14	2
	Scenario 1: current market	20%	119	41	105	237	305	9	18	1
		35%	**110***	**41***	**100***	**211***	282	10	21	1
		50%	**102***	**40***	**93***	**209***	262	10	**24***	0
		100%	**73***	**32***	**66***	**123***	221	**25***	**47***	0
	Scenario 2A: mandatory PBA fortification	20%	130	48	115	251	320	7	13	2
		35%	127	48	115	250	302	7	13	1
		50%	126	47	112	238	300	7	13	1
		100%	118	48	110	**202***	300	7	13	1
	Scenario 2B: mandatory bread iodisation	0%	**151***	**67***	**136***	282	373	2	**5***	3
		20%	145	60	129	279	371	3	7	3
		35%	132	60	121	244	313	3	8	2
		50%	121	57	111	**215***	302	3	9	1
		100%	**92***	44	**86***	**142***	233	8	23	1
	Scenario 3: comprehensive mandatory fortification	20%	**149***	**67***	**136***	274	349	2	4	3
		35%	144	**66***	**133***	256	332	2	5	3
		50%	141	61	129	258	327	2	5	3
		100%	137	**66***	129	221	311	3	7	3
**Females aged 4-8 years (n = 251)**	Usual diet	0%	115	53	104	226	261	5	14	0
	Scenario 1: current market	20%	105	49	94	196	245	6	16	0
		35%	**98***	44	**90***	169	228	8	20	0
		50%	**89***	41	**83***	**165***	**206***	11	27	0
		100%	**64***	**31***	**58***	**113***	**152***	**35***	**60***	0
	Scenario 2A: mandatory PBA fortification	20%	113	52	103	211	260	5	13	0
		35%	111	51	100	197	253	5	14	0

		% replacement of dairy	Mean	5th	50th	95th	99th	% < LRNI	% < EAR	% > UL
		50%	109	50	99	184	260	5	14	0
		100%	104	49	98	**169***	238	6	16	0
	Scenario 2B: mandatory bread iodisation	0%	**132***	**64***	**120***	231	286	1	6	1
		20%	127	61	114	229	284	2	7	1
		35%	113	56	105	190	244	3	10	0
		50%	107	56	100	184	229	3	10	0
		100%	**81***	44	**75***	**130***	**177***	9	**32***	0
	Scenario 3: comprehensive mandatory fortification	20%	**130***	**64***	**121***	228	277	1	**6***	0
		35%	127	**64***	**118***	206	271	1	**6***	0
		50%	123	57	112	224	266	2	**6***	0
		100%	121	64	117	194	243	2	7	0
**Males aged 9-13 years (n = 260)**	Usual diet	0%	132	55	114	274	340	10	21	0
	Scenario 1: current market	20%	121	48	104	244	311	11	25	0
		35%	**113***	49	**99***	209	288	15	28	0
		50%	**106***	43	**90***	**207***	306	**19***	31	0
		100%	**79***	**33***	**67***	**155***	229	**38***	**58***	0
	Scenario 2A: mandatory PBA fortification	20%	130	55	113	266	328	10	21	0
		35%	128	53	112	255	318	9	22	0
		50%	127	55	112	257	320	10	22	0
		100%	120	53	105	229	289	10	22	0
	Scenario 2B: mandatory bread iodisation	0%	**155***	**71***	**138***	304	383	2	6	1
		20%	149	**65***	132	294	375	3	9	1
		35%	138	**65***	123	253	346	2	10	0
		50%	130	**61***	117	237	305	5	13	0
		100%	**102***	50	**91***	**179***	276	13	27	0
	Scenario 3: comprehensive mandatory fortification	20%	**153***	**71***	**136***	296	383	**2***	**6***	0
		35%	149	**70***	**134***	280	353	**2***	**6***	0
		50%	144	**67***	127	273	364	**2***	**6***	0
		100%	143	**71***	129	266	324	**3***	**8***	0
**Females aged 9-13 years (n = 231)**	Usual diet	0%	116	48	99	241	310	16	27	0
	Scenario 1: current market	20%	108	45	94	228	293	17	30	0
		35%	102	43	87	203	253	22	33	0

		% replacement of dairy	Mean	5th	50th	95th	99th	% < LRNI	% < EAR	% > UL
		50%	**95***	41	**84***	194	250	23	38	0
		100%	**73***	**32***	**65***	**139***	**206***	**44***	**59***	0
	Scenario 2A: mandatory PBA fortification	20%	114	46	99	236	313	15	27	0
		35%	112	49	96	227	300	16	27	0
		50%	112	46	97	227	291	16	28	0
		100%	106	46	93	204	241	16	29	0
	Scenario 2B: mandatory bread iodisation	0%	**136***	**64***	**120***	256	326	**3***	**10***	0
		20%	130	58	116	247	320	**6***	**13***	0
		35%	121	**59***	111	231	270	**6***	16	0
		50%	115	58	106	202	264	**6***	17	0
		100%	**93***	45	**88***	**170***	220	16	32	0
	Scenario 3: comprehensive mandatory fortification	20%	**134***	**64***	**120***	254	328	**3***	**10***	0
		35%	131	**63***	119	240	305	**3***	**11***	0
		50%	126	59	113	240	326	**4***	**11***	0
		100%	126	63	114	214	268	**6***	**13***	0
**Males aged 14-18 years (n = 192)**	Usual diet	0%	133	42	110	287	395	20	39	0
	Scenario 1: current market	20%	124	41	107	259	364	22	44	0
		35%	116	41	98	247	328	25	47	0
		50%	**108***	39	92	224	295	28	52	0
		100%	**84***	31	**72***	**166***	**260***	**48***	**72***	0
	Scenario 2A: mandatory PBA fortification	20%	130	42	111	289	385	21	39	0
		35%	129	41	110	282	371	20	39	0
		50%	128	41	110	275	336	20	38	0
		100%	122	41	105	250	344	21	39	1
	Scenario 2B: mandatory bread iodisation	0%	**159***	61	**140***	338	402	10	23	1
		20%	152	53	130	323	396	12	27	0
		35%	142	56	126	285	374	13	31	0
		50%	133	50	119	263	325	14	29	0
		100%	**110***	43	100	**209***	284	22	46	0
	Scenario 3: comprehensive mandatory fortification	20%	157	61	138	331	405	**9***	**23***	1
		35%	154	58	138	306	398	10	**23***	1
		50%	147	55	128	300	392	10	**24***	1

		% replacement of dairy	Mean	5th	50th	95th	99th	% < LRNI	% < EAR	% > UL
		100%	149	58	136	279	398	11	27	0
**Females aged 14-18 years (n = 217)**	Usual diet	0%	97	37	90	298	233	31	54	0
	Scenario 1: current market	20%	91	36	84	181	211	36	61	0
		35%	88	32	81	178	210	38	65	0
		50%	**84***	31	**78***	152	197	41	65	0
		100%	**71***	26	**65***	**143***	**181***	**55***	**80***	0
	Scenario 2A: mandatory PBA fortification	20%	96	37	88	192	238	31	56	0
		35%	95	37	88	188	231	31	57	0
		50%	94	36	86	192	217	32	57	0
		100%	92	35	84	172	221	34	59	0
	Scenario 2B: mandatory bread iodisation	0%	**136***	52	**112***	225	254	**17***	**37***	0
		20%	**111***	47	**105***	216	252	22	**41***	0
		35%	**108***	43	103	198	230	20	46	0
		50%	103	41	96	193	227	23	50	0
		100%	**91***	40	85	160	197	33	59	0
	Scenario 3: comprehensive mandatory fortification	20%	**115***	51	110	210	355	**17***	**37***	0
		35%	**114***	50	110	201	257	**17***	**47***	0
		50%	**111***	49	107	200	261	19	43	0
		100%	108	46	105	199	250	21	43	0
**Males aged 19-49 years (n = 358)**	Usual diet	0%	163	52	152	313	442	10	20	0
	Scenario 1: current market	20%	154	47	142	301	427	12	22	0
		35%	147	47	137	278	429	12	25	0
		50%	**143***	45	**133***	272	426	13	26	0
		100%	**121***	**39***	**106***	**239***	394	**23***	**41***	0
	Scenario 2A: mandatory PBA fortification	20%	162	52	150	305	441	10	20	0
		35%	160	51	149	301	436	10	20	0
		50%	159	51	146	297	437	10	20	0
		100%	155	50	143	284	435	11	20	0
	Scenario 2B: mandatory bread iodisation	0%	**190***	**74***	**180***	338	473	10	22	0
		20%	**182***	69	171	338	466	5	13	0
		35%	175	67	163	311	432	6	15	0
		50%	168	66	157	308	427	7	15	0

		% replacement of dairy	Mean	5th	50th	95th	99th	% < LRNI	% < EAR	% > UL
		100%	148	55	135	269	401	10	22	0
	Scenario 3: comprehensive mandatory fortification	20%	**188***	**74***	**179***	331	446	**4***	12	0
		35%	186	**72***	**174***	328	446	**4***	13	0
		50%	179	67	170	324	446	5	13	0
		100%	**182***	**72***	**170***	313	439	6	13	0
**Females aged 19-49 years (n = 554)**	Usual diet	0%	131	49	117	241	332	14	32	1
	Scenario 1: current market	20%	124	47	112	229	332	18	35	1
		35%	**119***	44	**107***	219	332	**21***	**42***	1
		50%	**113***	43	**100***	**202***	315	**23***	**45***	1
		100%	**94***	**31***	**81***	**179***	311	**39***	**64***	1
	Scenario 2A: mandatory PBA fortification	20%	129	49	116	241	332	14	32	1
		35%	128	49	114	240	332	15	32	1
		50%	127	49	113	233	330	15	34	1
		100%	123	48	110	226	325	16	37	1
	Scenario 2B: mandatory bread iodisation	0%	**150***	66	136	264	349	**6***	**20***	1
		20%	**143***	62	131	253	343	**8***	**24***	1
		35%	137	59	126	240	340	9	27	1
		50%	131	56	119	227	338	11	29	1
		100%	**113***	47	**100***	**199***	349	**21***	**44***	1
	Scenario 3: comprehensive mandatory fortification	20%	**148***	66	135	261	349	**6***	**20***	1
		35%	**146***	65	133	254	349	**6***	**21***	1
		50%	**142***	64	130	248	342	**7***	**22***	1
		100%	141	62	128	248	342	**8***	26	1
**Males aged 50-64 years (n = 212)**	Usual diet	0%	183	76	170	366	509	4	14	0
	Scenario 1: current market	20%	171	70	158	329	493	5	17	0
		35%	164	64	146	320	477	8	20	0
		50%	**156***	63	**138***	305	435	8	20	0
		100%	**129***	**51***	**107***	286	395	**18***	**37***	0
	Scenario 2A: mandatory PBA fortification	20%	180	75	167	352	506	4	15	0
		35%	179	76	164	352	501	4	15	0
		50%	176	76	161	350	463	4	15	0
		100%	170	74	154	348	473	4	17	0

		% replacement of dairy	Mean	5th	50th	95th	99th	% < LRNI	% < EAR	% > UL
	Scenario 2B: mandatory bread iodisation	0%	**210***	91	195	404	572	2	6	1
		20%	201	84	184	396	554	3	7	0
		35%	192	81	177	381	491	2	7	0
		50%	184	80	168	369	489	2	9	0
		100%	**156***	67	**138***	306	432	6	18	0
	Scenario 3: comprehensive mandatory fortification	20%	207	91	191	394	565	2	6	0
		35%	204	89	189	383	543	1	7	0
		50%	197	83	182	377	530	1	7	0
		100%	197	86	177	388	499	2	8	0
**Females aged 50-64 years (n = 268)**	Usual diet	0%	157	62	143	286	403	7	18	0
	Scenario 1: current market	20%	145	59	134	269	362	9	23	0
		35%	**138***	54	**125***	251	332	11	25	0
		50%	**128***	50	**116***	240	329	14	**32***	0
		100%	**102***	**36***	**87***	**197***	273	**29***	**57***	0
	Scenario 2A: mandatory PBA fortification	20%	154	62	141	281	395	7	18	0
		35%	152	62	139	273	384	8	18	0
		50%	150	58	139	273	376	8	19	0
		100%	144	58	134	263	337	9	20	0
	Scenario 2B: mandatory bread iodisation	0%	**175***	68	**159***	312	416	6	10	0
		20%	168	68	152	299	407	6	12	0
		35%	157	64	143	279	380	6	13	0
		50%	147	63	137	263	340	8	18	0
		100%	**120***	47	**109***	**221***	286	15	**39***	0
	Scenario 3: comprehensive mandatory fortification	20%	172	66	**158***	307	414	5	11	0
		35%	168	69	157	302	385	5	11	0
		50%	165	66	152	292	392	6	13	0
		100%	162	65	152	288	346	6	13	0
**Males aged 65+ years (n = 193)**	Usual diet	0%	200	80	163	396	539	4	11	1
	Scenario 1: current market	20%	188	74	155	365	536	5	13	1
		35%	180	68	150	347	528	6	16	1
		50%	171	65	**135***	333	521	6	17	1
		100%	**141***	**49***	**115***	**270***	505	**17***	**36***	1

		% replacement of dairy	Mean	5th	50th	95th	99th	% < LRNI	% < EAR	% > UL
	Scenario 2A: mandatory PBA fortification	20%	197	77	161	390	542	4	10	1
		35%	195	79	157	383	545	4	10	1
		50%	193	73	156	373	542	4	10	1
		100%	186	70	149	343	549	5	11	1
	Scenario 2B: mandatory bread iodisation	0%	227	96	**196***	428	553	2	5	1
		20%	218	86	185	415	548	2	6	1
		35%	206	82	174	383	543	2	6	1
		50%	197	77	167	366	532	3	9	1
		100%	167	65	**141***	299	519	7	15	1
	Scenario 3: comprehensive mandatory fortification	20%	224	93	198	413	557	2	5	1
		35%	219	88	186	398	557	2	6	1
		50%	213	88	179	403	550	2	6	1
		100%	212	91	178	368	562	2	6	1
**Females aged 65+ years (n = 259)**	Usual diet	0%	150	62	142	282	339	8	20	0
	Scenario 1: current market	20%	140	58	130	259	327	10	23	0
		35%	**132***	55	**122***	257	298	12	27	0
		50%	**125***	51	**117***	240	300	14	**31***	0
		100%	**100***	**39***	**91***	**201***	280	**26***	**58***	0
	Scenario 2A: mandatory PBA fortification	20%	148	62	140	278	329	8	19	0
		35%	146	62	138	266	330	8	20	0
		50%	144	61	137	258	331	8	20	0
		100%	139	59	133	249	307	8	20	0
	Scenario 2B: mandatory bread iodisation	0%	**168***	79	**159***	298	354	4	11	0
		20%	161	75	152	290	350	4	12	0
		35%	152	72	143	279	341	5	16	0
		50%	143	65	134	256	328	7	18	0
		100%	**118***	50	**108***	222	299	16	35	0
	Scenario 3: comprehensive mandatory fortification	20%	**166***	80	**158***	288	352	4	**10***	0
		35%	163	75	156	284	349	4	12	0
		50%	159	69	152	284	343	5	12	0
		100%	158	74	151	277	332	5	13	0

* Indicates meaningfully different from usual diet based on non-overlapping confidence intervals

Supplementary Table 5. The impact of total or partial replacement of dairy milk, cheese, and yoghurt with plant-based alternatives on calcium (mg/day), riboflavin (mg/day) and vitamin B12 intakes and % with intakes below the EAR and LRNI

			**Calcium**					**Riboflavin**					**Vitamin B12**				
		**% replacement of dairy**	**Mean**	**5th**	**95th**	**% < LRNI**	**% < EAR**	**Mean**	**5th**	**95th**	**% < LRNI**	**% < EAR**	**Mean**	**5th**	**95th**	**% < LRNI**	**% < EAR**
**Males aged 1.5-3 years (n = 164)**	Usual diet	0%	746	383	1218	0	1	1.4	0.7	2.2	0	1	3.9	1.3	6.6	0	0
	Scenario 1: current market	20%	707	373	1156	0	2	1.2	0.6	2.1	0	1	3.5	1.3	6.0	0	0
		35%	677	368	1105	0	2	**1.2***	0.6	1.9	0	2	**3.3***	1.2	5.7	0	0
		50%	**660***	369	1025	0	2	**1.1***	0.6	**1.8**	0	2	**3.1***	1.1	**5.2***	0	0
		100%	**557***	309	**853***	0	4	**0.8***	**0.5***	**1.3***	0	7	**2.2***	1.0	**3.8***	0	0
	Scenario 2A: mandatory PBA fortification	20%	735	383	1220	0	1	1.3	0.6	2.2	0	1	3.7	1.2	6.3	0	0
		35%	728	382	1215	0	1	1.3	0.7	2.2	0	1	3.5	1.2	5.9	0	0
		50%	718	362	1213	0	1	1.3	0.6	2.2	0	1	**3.3***	1.2	5.7	0	0
		100%	693	340	1174	0	2	1.3	0.6	2.2	0	1	**2.7***	1.2	**4.8***	0	0
**Females aged 1.5-3 years (n = 142)**	Usual diet	0%	687	308	1033	1	2	1.3	0.5	2.0	0	4	3.6	1.4	6.3	0	0
	Scenario 1: current market	20%	653	303	1009	1	3	1.2	0.5	1.8	0	5	3.3	1.4	6.2	0	0
		35%	620	295	952	1	4	**1.1***	0.5	1.6	0	6	**3.0***	1.2	5.2	0	0
		50%	**601***	297	892	1	4	**1.0***	0.5	**1.6***	0	4	**2.8***	1.2	4.8	0	0
		100%	**515***	257	**822***	1	7	**0.8***	0.5	**1.2***	0	11	**2.1***	0.9	**3.6***	0	0
	Scenario 2A: mandatory PBA fortification	20%	677	309	1022	1	3	1.2	0.5	2.0	0	5	3.3	1.4	5.7	0	0
		35%	669	309	1022	1	2	1.2	0.5	1.9	0	4	3.2	1.3	5.7	0	0
		50%	660	306	1033	1	4	1.2	0.5	1.9	0	5	**3.1***	1.4	5.6	0	0
		100%	634	296	1034	1	4	1.2	0.5	1.9	0	5	**2.5***	1.1	**4.4***	0	0
**Males aged 4-8 years (n = 257)**	Usual diet	0%	767	378	1285	2	6	1.4	0.6	2.4	2	8	3.9	1.5	6.9	0	0
	Scenario 1: current market	20%	730	356	1234	2	8	1.3	0.6	2.2	2	9	3.6	1.4	6.2	0	0
		35%	708	363	1160	2	9	**1.3***	0.6	2.1	2	9	**3.4***	1.4	6.0	0	0
		50%	**681***	357	1120	2	10	**1.2***	0.6	**2.0***	2	11	**3.2***	1.3	5.8	0	1
		100%	**599***	325	**982***	4	14	**1.0***	**0.5***	**1.6***	4	**18***	**2.5***	**1.2***	**4.4***	0	2
	Scenario 2A: mandatory PBA fortification	20%	751	378	1269	2	6	1.4	0.6	2.3	2	8	3.7	1.4	6.6	0	0
		35%	743	370	1224	2	7	1.4	0.6	2.3	2	8	3.5	1.4	6.2	0	0
		50%	738	370	1251	2	7	1.4	0.6	2.3	2	8	**3.4***	1.4	6.1	0	0
		100%	703	359	1172	2	10	1.3	0.6	2.1	2	9	**2.9***	1.3	**4.9***	0	0
	Usual diet	0%	691	391	1098	1	5	1.2	0.6	2.0	1	9	3.5	1.5	6.3	0	0

			Calcium					Riboflavin					Vitamin B12				
		% replacement of dairy	Mean	5th	95th	% < LRNI	% < EAR	Mean	5th	95th	% < LRNI	% < EAR	Mean	5th	95th	% < LRNI	% < EAR
**Females aged 4-8 years (n = 251)**	Scenario 1: current market	20%	663	384	1051	1	6	1.2	0.6	1.8	2	11	3.3	1.4	5.8	0	0
		35%	642	371	1023	2	7	**1.1***	0.6	1.7	1	13	**3.1***	1.5	5.6	0	0
		50%	**614***	356	959	2	10	**1.0***	0.5	1.7	2	14	**2.9***	1.2	5.4	0	0
		100%	**543***	**322***	**874***	3	**18***	**0.9***	**0.5***	**1.3***	4	**29***	**2.3***	1.1	**4.4***	0	2
	Scenario 2A: mandatory PBA fortification	20%	682	391	1097	1	5	1.2	0.6	2.0	1	9	3.4	1.4	5.9	0	0
		35%	671	383	1103	2	6	1.2	0.6	2.0	1	10	3.2	1.4	5.9	0	0
		50%	661	370	1084	2	8	1.2	0.6	1.9	1	10	3.1	1.4	5.5	0	1
		100%	633	363	1019	2	10	1.2	0.6	1.9	1	10	**2.7***	1.3	**5.1***	0	1
**Males aged 9-13 years (n = 260)**	Usual diet	0%	828	397	1391	5	8	1.5	0.7	2.7	4	15	4.4	1.8	8.3	0	0
	Scenario 1: current market	20%	796	384	1312	6	9	1.5	0.6	2.5	6	18	4.1	1.7	7.5	0	0
		35%	771	384	1259	6	9	1.4	0.6	2.4	6	20	**3.9***	1.6	7.0	0	1
		50%	749	387	1224	7	11	**1.3***	0.6	2.4	7	21	**3.7***	1.5	**6.8***	1	1
		100%	**668***	352	1039	10	14	**1.1***	0.5	**1.9***	11	**31***	**3.1***	1.3	**5.4***	1	1
	Scenario 2A: mandatory PBA fortification	20%	815	397	1367	5	9	1.5	0.7	2.7	4	16	4.2	1.7	7.8	0	0
		35%	8.7	397	1368	6	9	1.5	0.7	2.6	4	16	4.1	1.7	7.5	0	0
		50%	796	397	1349	5	10	1.5	0.7	2.6	5	16	3.9	1.6	6.9	1	1
		100%	762	376	1268	7	11	1.5	0.7	2.6	5	16	**3.5***	1.5	**6.0***	1	1
**Females aged 9-13 years (n = 231)**	Usual diet	0%	707	390	1139	7	12	1.3	0.6	2.2	7	24	3.7	1.5	6.6	0	0
	Scenario 1: current market	20%	682	386	1082	7	13	1.2	0.6	2.0	7	26	3.5	1.5	6.2	0	0
		35%	661	377	1038	9	14	1.2	0.6	2.0	10	30	3.4	1.5	5.8	0	0
		50%	637	361	990	10	18	**1.1***	0.6	1.8	10	35	**3.2***	1.5	**5.5***	0	0
		100%	**571***	330	**879***	**16***	**24***	**0.9***	0.5	**1.5***	**17***	**49***	**2.7***	1.3	**4.4***	0	2
	Scenario 2A: mandatory PBA fortification	20%	692	342	1047	8	13	1.3	0.6	2.1	7	25	3.6	1.7	6.1	0	0
		35%	683	379	1084	7	14	1.3	0.6	2.1	7	26	3.4	1.7	6.0	0	0
		50%	675	389	1091	9	15	1.2	0.6	2.1	7	26	3.4	1.6	5.9	0	0
		100%	**643***	377	1097	13	19	1.2	0.6	2.1	8	26	**3.0***	1.5	**5.1***	0	0
**Males aged 14-18 years (n = 192)**	Usual diet	0%	842	339	1496	17	30	1.6	0.6	3.1	13	21	4.8	1.4	9.2	2	2
	Scenario 1: current market	20%	809	338	1418	18	33	1.5	0.6	2.9	16	22	4.5	1.4	8.8	2	3
		35%	784	338	1394	19	35	1.5	0.6	2.7	17	24	4.3	1.4	8.3	2	3
		50%	761	318	1331	19	36	1.4	0.6	2.6	17	24	4.2	1.4	8.0	2	4
		100%	**681***	307	**1205***	25	**47***	**1.2***	0.5	**2.3***	24	**33***	**3.6***	1.1	**6.8***	4	6

		% replacement of dairy	Calcium					Riboflavin					Vitamin B12				
			Mean	5th	95th	% < LRNI	% < EAR	Mean	5th	95th	% < LRNI	% < EAR	Mean	5th	95th	% < LRNI	% < EAR
	Scenario 2A: mandatory PBA fortification	20%	825	339	1479	16	32	1.6	0.6	3.1	13	21	4.6	1.4	9.0	2	2
		35%	818	321	1482	17	32	1.6	0.6	3.1	13	21	4.5	1.4	8.6	2	3
		50%	808	321	1445	16	33	1.6	0.6	3.0	14	21	4.3	1.3	8.2	2	5
		100%	768	321	1345	20	38	1.5	0.6	3.0	15	22	**3.9***	1.2	7.4	4	6
**Females aged 14-18 years (n = 217)**	Usual diet	0%	641	283	1041	25	54	1.2	0.5	2.1	24	30	3.6	1.3	7.5	2	5
	Scenario 1: current market	20%	618	282	1003	28	56	1.1	0.5	2.0	25	35	3.4	1.3	7.3	2	5
		35%	607	281	986	30	59	1.1	0.5	1.9	25	36	3.3	1.2	7.2	3	6
		50%	588	252	939	31	63	1.1	0.4	1.9	28	39	3.2	1.1	6.8	3	7
		100%	**537***	251	**838***	**44***	**71***	**1.0***	0.4	**1.7***	**36***	**52***	**2.9***	1.0	6.2	5	11
	Scenario 2A: mandatory PBA fortification	20%	629	283	1022	26	55	1.1	0.5	2.1	24	32	3.5	1.2	7.3	4	5
		35%	621	283	1005	27	56	1.1	0.5	2.1	24	32	3.4	1.1	7.0	3	6
		50%	614	275	993	28	56	1.1	0.5	2.1	24	32	3.3	1.2	6.9	3	6
		100%	582	275	927	32	64	1.1	0.5	2.0	25	35	3.1	1.1	6.6	4	7
**Males aged 19-49 years (n = 358)**	Usual diet	0%	888	410	1543	5	13	1.7	0.8	2.9	6	14	5.4	1.8	10.5	1	2
	Scenario 1: current market	20%	851	403	1430	5	15	1.6	0.8	2.8	7	15	5.1	1.6	10.4	1	2
		35%	829	402	1398	5	16	1.6	0.7	2.7	8	16	5.0	1.6	9.7	1	3
		50%	805	390	**1332***	6	17	**1.6***	0.7	2.6	9	18	4.8	1.6	9.7	2	4
		100%	**719***	357	**1164***	8	**21***	**1.4***	**0.7***	**2.4***	**13***	**26***	**4.3***	**1.2***	9.1	3	6
	Scenario 2A: mandatory PBA fortification	20%	869	402	1514	5	14	1.7	0.8	2.9	6	14	5.2	1.6	10.1	1	2
		35%	850	401	1436	5	15	1.7	0.8	2.9	7	15	5.1	1.6	10.1	1	3
		50%	845	402	1463	5	15	1.7	0.8	2.9	7	15	5.0	1.6	10.1	1	3
		100%	**793***	387	**1316***	6	17	1.7	0.7	2.9	8	15	**4.6***	1.4	9.4	3	4
**Females aged 19-49 years (n = 554)**	Usual diet	0%	727	341	1273	10	26	1.4	0.6	2.4	14	29	4.4	1.5	8.1	2	4
	Scenario 1: current market	20%	699	327	1219	12	27	1.3	0.6	2.3	16	31	4.2	1.4	7.8	3	5
		35%	**677***	320	1155	12	30	1.3	0.6	2.2	17	35	4.1	1.3	7.8	3	5
		50%	**653***	315	**1102***	13	33	**1.2***	0.6	**2.1***	20	**39***	3.9	1.2	7.6	3	5
		100%	**580***	288	**960***	**19***	**46***	**1.1***	**0.5***	**1.9***	**28***	**50***	**3.4***	**1.0***	7.0	6	9
	Scenario 2A: mandatory PBA fortification	20%	709	335	1232	10	27	1.4	1.3	2.4	14	29	4.2	1.4	7.9	3	4
		35%	701	330	1226	11	27	1.3	1.3	2.4	15	29	4.1	1.4	7.8	3	4
		50%	684	324	1190	11	29	1.3	1.3	2.4	15	30	4.0	1.3	7.5	3	5
		100%	**642***	310	**1066***	13	35	1.3	1.2	2.3	16	31	3.7	1.1	7.4	5	7

		% replacement of dairy	Calcium					Riboflavin					Vitamin B12				
			Mean	5th	95th	% < LRNI	% < EAR	Mean	5th	95th	% < LRNI	% < EAR	Mean	5th	95th	% < LRNI	% < EAR
**Males aged 50-64 years (n = 212)**	Usual diet	0%	864	413	1386	5	15	1.7	0.9	3.0	4	13	5.5	2.0	10.4	0	2
	Scenario 1: current market	20%	827	406	1314	5	17	1.7	0.8	2.8	5	15	5.3	2.0	10.2	1	2
		35%	805	379	1296	6	16	1.6	0.8	2.9	6	17	5.1	1.9	9.9	1	2
		50%	**777***	376	1266	7	18	**1.5***	0.8	2.7	6	17	4.9	1.7	9.6	2	2
		100%	**691***	348	1134	9	22	**1.3***	0.7	2.4	**11***	**27***	**4.2***	1.3	9.3	3	4
	Scenario 2A: mandatory PBA fortification	20%	848	412	1327	4	15	1.7	0.9	3.0	4	13	5.3	1.9	10.1	1	2
		35%	838	411	1319	4	15	1.7	0.8	3.0	4	14	5.2	1.8	9.9	1	2
		50%	825	403	1333	5	16	1.7	0.8	3.0	4	14	5.0	1.8	9.8	2	2
		100%	785	393	1279	6	18	1.7	0.8	2.9	4	15	**4.6***	1.5	9.5	2	3
**Females aged 50-64 years (n = 268)**	Usual diet	0%	774	352	1269	8	21	1.5	1.5	2.4	8	16	4.8	1.9	9.1	1	2
	Scenario 1: current market	20%	740	342	1170	9	22	1.4	1.4	2.3	9	19	4.6	1.7	8.9	2	3
		35%	716	339	1157	10	24	**1.4***	1.3	2.2	11	20	4.4	1.7	8.6	3	3
		50%	**688***	322	1110	12	27	**1.3***	1.3	2.1	13	24	**4.2***	1.4	8.4	3	3
		100%	**603***	307	**924***	**16***	**37***	**1.1***	1.1	**1.7***	**17***	**41***	**3.6***	**1.1***	7.8	**5***	**8***
	Scenario 2A: mandatory PBA fortification	20%	762	354	1246	8	22	1.5	0.7	2.4	8	17	4.7	1.8	8.9	2	3
		35%	747	342	1219	9	23	1.5	0.7	2.4	8	17	4.5	1.7	8.5	2	3
		50%	734	342	1185	9	23	1.5	0.7	2.4	8	17	4.4	1.5	8.5	2	3
		100%	695	332	1146	10	26	1.5	0.7	2.4	9	18	**3.9***	1.4	8.2	3	4
**Males aged 65+ years (n = 193)**	Usual diet	0%	863	421	1471	5	13	1.8	0.9	3.1	5	8	6.4	2.2	13.5	1	2
	Scenario 1: current market	20%	829	401	1439	5	15	1.7	0.9	2.7	5	10	6.1	2.2	13.3	1	2
		35%	799	399	1362	6	17	1.6	0.8	2.7	5	11	5.9	2.1	13.0	2	2
		50%	**772***	392	1300	6	18	**1.6***	0.8	2.5	5	12	5.6	2.0	12.9	2	3
		100%	**681***	367	**1116***	10	**25***	**1.4***	0.7	**2.3***	8	**24***	**4.9***	1.8	12.2	2	3
	Scenario 2A: mandatory PBA fortification	20%	847	417	1457	5	14	1.8	0.9	3.1	5	8	6.2	2.1	13.4	1	2
		35%	837	408	1459	5	15	1.8	0.9	3.0	5	9	6.0	2.1	13.1	1	2
		50%	827	419	1428	5	16	1.8	0.9	3.0	5	9	5.8	2.0	13.0	2	2
		100%	783	395	1395	6	19	1.7	0.9	2.9	5	9	5.3	2.0	12.6	2	3
**Females aged 65+ years**	Usual diet	0%	733	348	1144	8	20	1.4	0.7	2.3	10	17	4.6	1.8	7.8	1	2
	Scenario 1: current market	20%	703	323	1118	9	22	1.3	0.7	2.2	11	21	4.4	1.6	7.6	1	2
		35%	675	319	1061	10	28	**1.3***	0.6	2.1	13	25	4.2	1.5	7.2	2	3
		50%	654	316	**1006***	10	**32***	**1.2***	0.6	**2.0***	12	**28***	4.0	1.4	7.4	2	4

			Calcium					Riboflavin					Vitamin B12				
		**% replacement of dairy**	**Mean**	**5th**	**95th**	**% < LRNI**	**% < EAR**	**Mean**	**5th**	**95th**	**% < LRNI**	**% < EAR**	**Mean**	**5th**	**95th**	**% < LRNI**	**% < EAR**
**(n = 259)**		100%	**574***	291	**867***	**18***	**46***	**1.0***	0.5	**1.7***	**25***	**50***	**3.4***	**1.1***	6.7	3	7
	Scenario 2A: mandatory PBA fortification	20%	719	348	1137	8	22	1.4	0.7	2.3	11	18	4.4	1.7	7.7	2	2
		35%	707	348	1105	8	22	1.4	0.7	2.2	10	17	4.3	1.6	7.5	2	2
		50%	698	347	1084	9	25	1.4	0.7	2.2	11	19	4.2	1.5	7.3	2	3
		100%	**663***	329	**1049***	10	31	1.4	0.7	2.2	11	20	3.8	1.4	6.9	2	4

Supplementary Table 6. The impact of total or partial replacement of dairy milk, cheese, and yoghurt with plant-based alternatives and/or the addition of iodised salt to bread products (20 mg of iodine per kg of salt) on iodine intakes (µg/day) and % with intakes below the EAR and LRNI and above the UL.

		% replacement of dairy	Mean	5th	50th	95th	99th	% < LRNI	% < EAR	% > UL
Males aged 1.5-3 years (n = 164)	Usual diet	0%	134	45	116	260	295	3	16	20
	Scenario 2B: mandatory bread iodisation (20 mg/kg of salt)	0%	143	56	129	269	335	1	13	23
		20%	138	49	126	258	295	1	15	21
		35%	118	46	109	216	248	3	16	10
		50%	104	42	99	184	211	5	21	2
		100%	70	32	65	119	144	13	50	0
	Scenario 3: comprehensive mandatory fortification	20%	139	56	129	257	285	1	12	19
		35%	137	53	129	259	291	1	13	18
		50%	135	55	127	246	290	1	13	15
		100%	128	50	125	222	284	1	13	10
Females aged 1.5-3 years (n = 142)	Usual diet	0%	121	48	107	238	295	4	15	9
	Scenario 2B: mandatory bread iodisation	0%	130	53	119	244	311	2	9	11
		20%	125	51	116	240	303	3	10	9
		35%	106	47	94	198	259	2	13	4
		50%	98	45	89	180	238	3	18	2
		100%	65	33	64	104	139	10	46	0
	Scenario 3: comprehensive mandatory fortification	20%	126	52	116	227	293	2	9	10
		35%	125	53	117	221	281	2	10	10
		50%	124	52	119	216	269	2	10	9
		100%	117	52	108	196	238	2	9	4
Males aged 4-8 years (n = 257)	Usual diet	0%	132	48	117	265	342	7	14	2
	Scenario 2B: mandatory bread iodisation	0%	146	62	130	280	363	4	10	4
		20%	141	55	128	272	352	5	14	4
		35%	124	56	112	223	303	6	15	2
		50%	117	54	106	208	275	6	20	1
		100%	86	42	81	137	229	18	45	1
	Scenario 3: comprehensive mandatory fortification	20%	143	62	128	264	338	4	10	4
		35%	140	61	127	251	327	4	10	4
		50%	138	61	127	250	310	4	10	3
		100%	131	60	124	213	307	4	12	3

**Females aged 4-8 years (n = 251)**	**Usual diet**	**0%**	**115**	**53**	**104**	**226**	**261**	**5**	**14**	**0**
	**Scenario 2B: mandatory bread iodisation**	**0%**	**128**	**58**	**115**	**234**	**289**	**3**	**12**	**1**
		**20%**	**123**	**57**	**111**	**229**	**274**	**4**	**15**	**1**
		**35%**	**109**	**55**	**101**	**190**	**226**	**4**	**15**	**0**
		**50%**	**101**	**53**	**95**	**170**	**223**	**5**	**22**	**0**
		**100%**	**76**	**40**	**70**	**123**	**168**	**22**	**63**	**0**
	**Scenario 3: comprehensive mandatory fortification**	**20%**	**125**	**58**	**114**	**213**	**271**	**4**	**11**	**1**
		**35%**	**123**	**59**	**111**	**207**	**270**	**3**	**11**	**1**
		**50%**	**122**	**59**	**113**	**208**	**258**	**2**	**11**	**1**
		**100%**	**116**	**57**	**110**	**185**	**242**	**4**	**12**	**0**
**Males aged 9-13 years (n = 260)**	**Usual diet**	**0%**	**132**	**55**	**114**	**274**	**340**	**10**	**21**	**0**
	**Scenario 2B: mandatory bread iodisation**	**0%**	**148**	**67**	**131**	**295**	**368**	**5**	**14**	**1**
		**20%**	**142**	**63**	**127**	**284**	**363**	**7**	**20**	**1**
		**35%**	**130**	**60**	**114**	**250**	**319**	**9**	**23**	**1**
		**50%**	**120**	**58**	**106**	**222**	**285**	**12**	**25**	**1**
		**100%**	**95**	**46**	**84**	**171**	**259**	**29**	**54**	**1**
	**Scenario 3: comprehensive mandatory fortification**	**20%**	**145**	**66**	**130**	**279**	**347**	**5**	**15**	**1**
		**35%**	**144**	**64**	**128**	**274**	**356**	**4**	**15**	**1**
		**50%**	**141**	**66**	**128**	**259**	**334**	**4**	**15**	**1**
		**100%**	**136**	**66**	**121**	**255**	**307**	**5**	**15**	**1**
**Females aged 9-13 years (n = 231)**	**Usual diet**	**0%**	**116**	**48**	**99**	**241**	**310**	**16**	**27**	**0**
	**Scenario 2B: mandatory bread iodisation**	**0%**	**130**	**60**	**113**	**250**	**339**	**8**	**19**	**0**
		**20%**	**125**	**56**	**110**	**246**	**313**	**12**	**24**	**0**
		**35%**	**115**	**54**	**104**	**216**	**262**	**13**	**24**	**0**
		**50%**	**108**	**50**	**98**	**205**	**270**	**20**	**35**	**0**
		**100%**	**87**	**40**	**81**	**158**	**214**	**34**	**57**	**0**
	**Scenario 3: comprehensive mandatory fortification**	**20%**	**127**	**60**	**113**	**244**	**313**	**8**	**20**	**0**
		**35%**	**126**	**60**	**112**	**238**	**305**	**8**	**19**	**0**
		**50%**	**125**	**60**	**112**	**238**	**300**	**7**	**20**	**0**
		**100%**	**119**	**58**	**110**	**211**	**257**	**8**	**22**	**0**
**Males aged 14-18 years (n = 192)**	**Usual diet**	**0%**	**133**	**42**	**110**	**287**	**395**	**20**	**39**	**0**
	**Scenario 2B: mandatory bread iodisation**	**0%**	**151**	**57**	**131**	**318**	**401**	**14**	**30**	**0**
		**20%**	**146**	**53**	**125**	**314**	**403**	**16**	**34**	**0**
		**35%**	**134**	**51**	**118**	**285**	**374**	**18**	**38**	**0**
		**50%**	**125**	**50**	**112**	**261**	**306**	**21**	**41**	**0**

		100%	102	40	91	193	276	31	61	0
	Scenario 3: comprehensive mandatory fortification	20%	149	56	131	314	391	14	30	0
		35%	147	56	130	308	378	15	31	0
		50%	145	56	130	285	396	15	29	1
		100%	140	55	127	270	382	16	30	1
Females aged 14-18 years (n = 217)	Usual diet	0%	97	37	90	298	233	31	54	0
	Scenario 2B: mandatory bread iodisation	0%	110	46	105	214	246	28	55	0
		20%	106	43	99	210	242	30	60	0
		35%	101	44	95	199	222	36	66	0
		50%	97	39	89	179	218	38	70	0
		100%	85	36	79	157	188	51	86	0
	Scenario 3: comprehensive mandatory fortification	20%	109	46	105	207	248	28	56	0
		35%	108	47	103	201	248	28	55	0
		50%	108	47	103	198	254	29	55	0
		100%	105	44	100	187	251	29	59	0
Males aged 19-49 years (n = 358)	Usual diet	0%	163	52	152	313	442	10	20	0
	Scenario 2B: mandatory bread iodisation	0%	185	67	172	340	498	14	28	0
		20%	176	61	165	319	456	16	32	0
		35%	167	60	155	302	441	17	34	0
		50%	161	57	150	287	429	18	37	0
		100%	140	50	126	261	399	27	57	0
	Scenario 3: comprehensive mandatory fortification	20%	180	67	168	320	449	15	29	0
		35%	179	67	169	321	444	14	27	0
		50%	178	67	167	314	445	14	29	0
		100%	173	67	162	300	438	15	30	0
Females aged 19-49 years (n = 554)	Usual diet	0%	131	49	117	241	332	14	32	1
	Scenario 2B: mandatory bread iodisation	0%	145	62	130	267	371	27	81	2
		20%	139	60	126	247	341	32	89	2
		35%	131	53	120	240	324	38	103	2
		50%	126	53	112	221	338	50	111	2
		100%	107	43	94	189	320	88	173	2
	Scenario 3: comprehensive mandatory fortification	20%	142	62	129	255	341	26	84	2
		35%	141	62	128	243	341	26	87	2
		50%	140	60	127	244	341	27	89	2
		100%	136	58	125	240	333	30	91	2

**Males aged 50-64 years (n = 212)**	**Usual diet**	**0%**	**183**	**76**	**170**	**366**	**509**	**4**	**14**	**0**
	**Scenario 2B: mandatory bread iodisation**	**0%**	**206**	**84**	**190**	**415**	**568**	**4**	**9**	**1**
		**20%**	**195**	**80**	**178**	**382**	**541**	**4**	**13**	**1**
		**35%**	**182**	**76**	**163**	**362**	**480**	**6**	**12**	**0**
		**50%**	**174**	**77**	**157**	**335**	**431**	**5**	**14**	**0**
		**100%**	**148**	**62**	**126**	**299**	**419**	**10**	**29**	**0**
	**Scenario 3: comprehensive mandatory fortification**	**20%**	**199**	**84**	**182**	**382**	**548**	**3**	**10**	**0**
		**35%**	**197**	**83**	**181**	**379**	**551**	**4**	**10**	**0**
		**50%**	**195**	**83**	**177**	**377**	**534**	**4**	**10**	**0**
		**100%**	**189**	**81**	**167**	**381**	**491**	**2**	**10**	**0**
**Females aged 50-64 years (n = 268)**	**Usual diet**	**0%**	**157**	**62**	**143**	**286**	**403**	**7**	**18**	**0**
	**Scenario 2B: mandatory bread iodisation**	**0%**	**171**	**66**	**155**	**304**	**435**	**10**	**20**	**1**
		**20%**	**164**	**65**	**151**	**297**	**407**	**10**	**22**	**0**
		**35%**	**149**	**62**	**137**	**276**	**355**	**10**	**30**	**0**
		**50%**	**141**	**59**	**128**	**258**	**337**	**13**	**35**	**0**
		**100%**	**114**	**44**	**101**	**212**	**279**	**27**	**71**	**0**
	**Scenario 3: comprehensive mandatory fortification**	**20%**	**167**	**66**	**153**	**299**	**404**	**10**	**21**	**0**
		**35%**	**165**	**67**	**152**	**299**	**386**	**10**	**21**	**0**
		**50%**	**162**	**64**	**150**	**287**	**389**	**10**	**21**	**0**
		**100%**	**156**	**63**	**145**	**281**	**343**	**10**	**24**	**0**
**Males aged 65+ years (n = 193)**	**Usual diet**	**0%**	**200**	**80**	**163**	**396**	**539**	**4**	**11**	**1**
	**Scenario 2B: mandatory bread iodisation**	**0%**	**220**	**87**	**185**	**417**	**548**	**2**	**7**	**1**
		**20%**	**212**	**84**	**181**	**402**	**547**	**2**	**7**	**1**
		**35%**	**197**	**79**	**164**	**374**	**534**	**4**	**13**	**1**
		**50%**	**190**	**72**	**160**	**355**	**542**	**4**	**12**	**1**
		**100%**	**159**	**61**	**133**	**293**	**515**	**10**	**24**	**1**
	**Scenario 3: comprehensive mandatory fortification**	**20%**	**216**	**87**	**181**	**413**	**550**	**2**	**7**	**1**
		**35%**	**213**	**88**	**180**	**402**	**550**	**3**	**7**	**1**
		**50%**	**211**	**88**	**178**	**396**	**553**	**2**	**9**	**1**
		**100%**	**203**	**87**	**167**	**360**	**558**	**2**	**9**	**1**
**Females aged 65+ years (n = 259)**	**Usual diet**	**0%**	**150**	**62**	**142**	**282**	**339**	**8**	**20**	**0**
	**Scenario 2B: mandatory bread iodisation**	**0%**	**167**	**70**	**155**	**299**	**368**	**8**	**18**	**1**
		**20%**	**157**	**68**	**149**	**284**	**343**	**9**	**23**	**0**
		**35%**	**144**	**64**	**134**	**269**	**324**	**10**	**30**	**0**
		**50%**	**136**	**62**	**127**	**245**	**305**	**13**	**35**	**0**

		**100%**	**112**	**45**	**103**	**215**	**294**	**27**	**66**	**0**
	**Scenario 3: comprehensive mandatory fortification**	**20%**	**160**	**71**	**154**	**284**	**344**	**8**	**19**	**0**
		**35%**	**158**	**72**	**151**	**287**	**340**	**8**	**20**	**0**
		**50%**	**157**	**70**	**149**	**280**	**342**	**9**	**19**	**0**
		**100%**	**152**	**69**	**145**	**266**	**326**	**9**	**21**	**0**

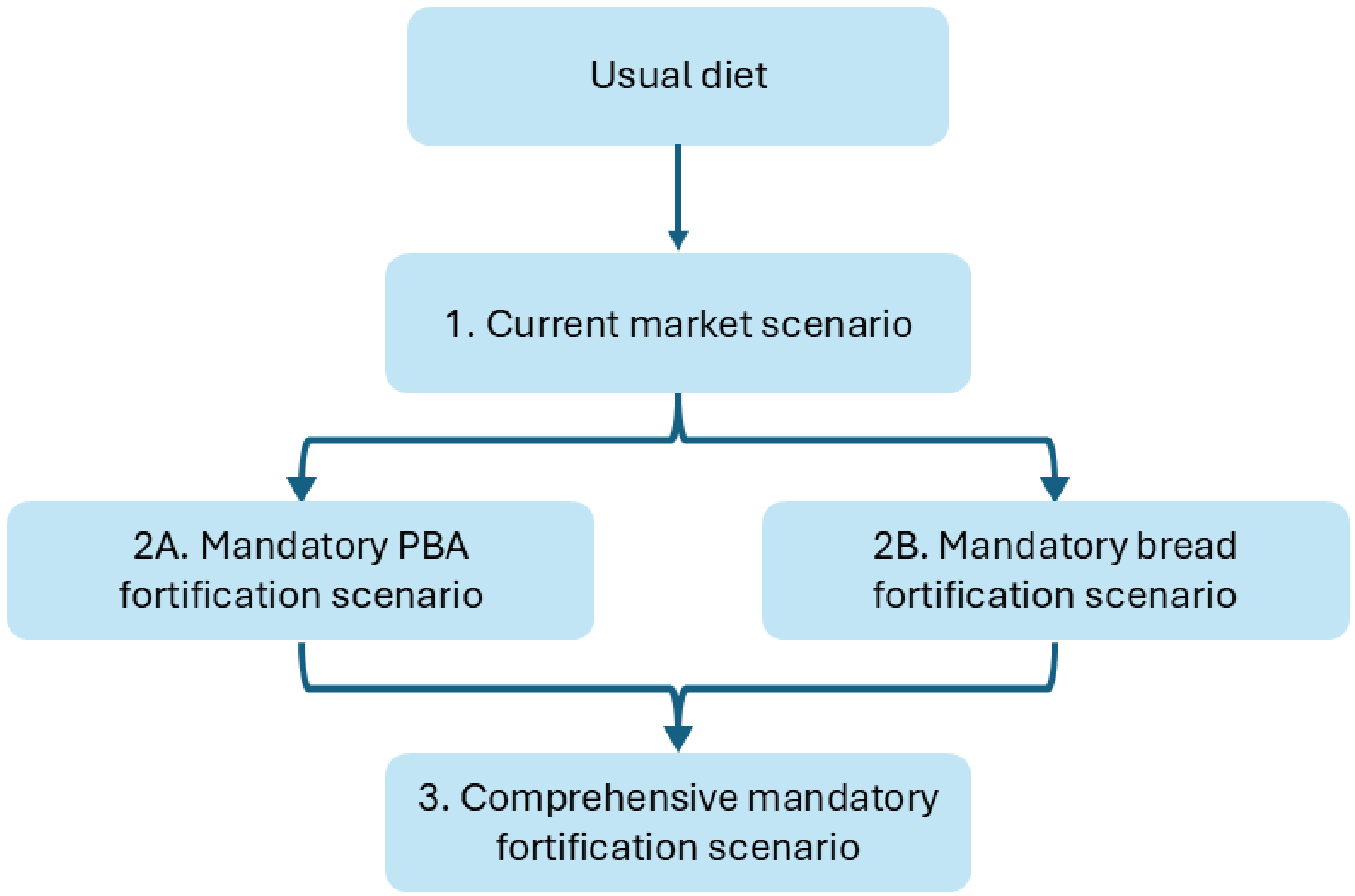

Supplementary Figure 1. Overview of dietary modelling scenarios.

Scenario 1: Partial replacement of dairy products with PBAs based on existing UK market fortification strategies (Current market fortification)
Scenario 2A: Partial replacement of dairy products with PBAs, assuming mandatory iodine fortification of PBAs at an industry-standard level (Mandatory PBA fortification)
Scenario 2B: Partial replacement of dairy products with PBAs based on existing fortification strategies, with mandatory iodine fortification of bread (Mandatory bread fortification)
Scenario 3: Partial replacement of dairy products with PBAs, assuming mandatory iodine fortification of both PBAs and bread (Comprehensive mandatory fortification)
